# Gene–Environment Interactions in Preventive Medicine: Current Status and Expectations for the Future

**DOI:** 10.3390/ijms18020302

**Published:** 2017-01-30

**Authors:** Hiroto Narimatsu

**Affiliations:** Cancer Prevention and Control Division, Kanagawa Cancer Center Research Institute, Yokohama 241-8515, Japan; hiroto-narimatsu@umin.org; Tel.: +81-45-520-2222

**Keywords:** personalized medicine, genome, prospective cohort study, precision medicine

## Abstract

The progression of many common disorders involves a complex interplay of multiple factors, including numerous different genes and environmental factors. Gene–environmental cohort studies focus on the identification of risk factors that cannot be discovered by conventional epidemiological methodologies. Such epidemiological methodologies preclude precise predictions, because the exact risk factors can be revealed only after detailed analyses of the interactions among multiple factors, that is, between genes and environmental factors. To date, these cohort studies have reported some promising results. However, the findings do not yet have sufficient clinical significance for the development of precise, personalized preventive medicine. Especially, some promising preliminary studies have been conducted in terms of the prevention of obesity. Large-scale validation studies of those preliminary studies, using a prospective cohort design and long follow-ups, will produce useful and practical evidence for the development of preventive medicine in the future.

## 1. Introduction

**Figure 1:**
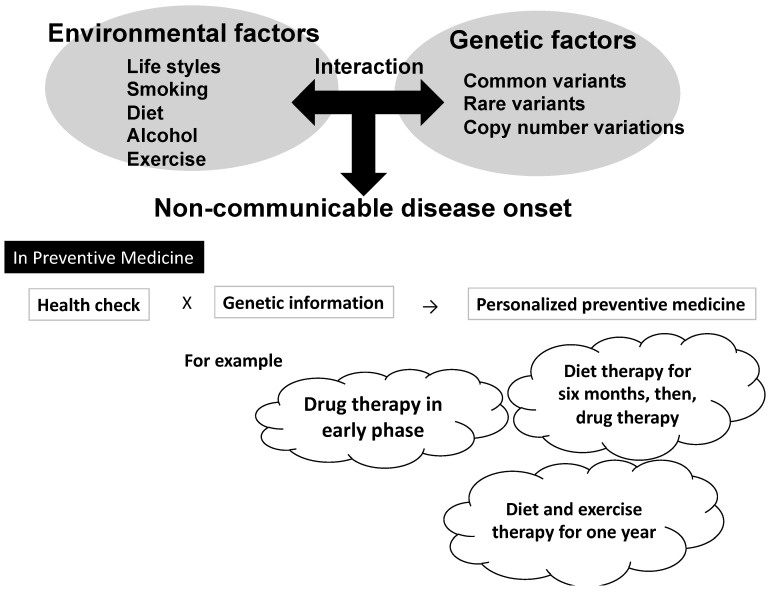
Concept of personalized preventive medicine using gene–environmental interaction.

The development of many common disorders involves complex interplays of multiple factors, including numerous genes and environmental factors. Gene–environmental cohort studies focus on the identification of risk factors that cannot be discovered by conventional epidemiological methodologies [1,2]. Such epidemiological methodologies preclude precise predictions, because the exact risk factors can be revealed only after detailed analyses of the interactions among multiple factors, that is, between different genes and environmental factors. To date, the cohort studies on gene–environmental interactions have reported some promising results. However, the findings do not yet have sufficient clinical significance to allow for the development of precise or personalized preventive medicine (Figure 1). In this paper, I review the findings of the available studies on gene–environmental interactions and discuss how to apply these findings to the development of preventive medicine.

## 2. Current Concepts and Study Design for Investigating Gene–Environmental Interactions

### 2.1. Current Concepts

To clarify gene–environmental interactions in non-communicable diseases (NCDs) is important in the field of preventive medicine. The genomic information from germ cell lines remains the same throughout the lifespan of a person. Thus, preventive medicine for such genomic factors cannot be established. On the other hand, environmental factors, particularly including personal lifestyle habits such as diet and exercise, can be improved by personal efforts. Thus, preventive interventions aimed at improving such factors are possible, and primary prevention against developing a disease in the first place by improving personal lifestyle habits will continue to be the focus of preventive medicine, even in the genomic medicine era. Nevertheless, information on gene–environmental interactions has the potential to enable precise disease prevention, and represents a major advantage in the genomic medicine era.

One of the advantages of obtaining information of gene–environmental interactions is the identification of target lifestyles at which the intervention should be aimed towards. Large*-*scale genomic studies, such as genome-wide association studies (GWASs), can only identify the association of genomic variations and the onset of NCDs [3]. However, information on the appropriate preventive interventions cannot be provided by such studies. Thus, only general interventions in diet and exercise, such as the implementation of low-calorie diets and high-intensity exercise programs, would be conducted in individuals with the high-risk genetic variations identified by GWASs.

In contrast, analyses of gene–environmental interactions can provide more practical and precise information. For example, clarification of the combinations of certain genetic variations and the exact nutritional intakes that increase or decrease the risk of disease onset would allow us to conduct more specific intervention programs, tailored to each individual, by recommending reduced or increased intake of a particular food or nutrient. Accordingly, compared to general instructions for diet or exercise therapy, more specific instructions based on the result of gene–environmental interaction studies would be more effective in the prevention of the disease.

### 2.2. Study Designs for Investigating Gene–Environmental Interactions

Recent GWASs have adopted a case-control design [3]. In such studies, the frequency of genomic variations was compared between the case and control groups. However, the risk of serious bias is substantial in gene–environmental interaction analyses. In case-control studies, DNA samples can be collected even after disease onset, because DNA information from germ cell lines remains unchanged throughout an individual’s lifespan. In contrast, environmental information must be collected before disease onset in order to clarify the causation of the disease. In gene–environmental interaction analyses, large amounts of information on lifestyle habits need to be collected [4,5,6], and it is always difficult to recall information from before the disease onset, especially in cases where the disease first developed 20 or 30 years ago. Even for relatively simple factors such as smoking habits, recall bias can be problematic [1].

Studies using a prospective cohort design have advantages related to the conduction of gene–environmental interaction analyses. However, a weakness of such studies is that they are associated with long follow-up periods and high costs [1]. Studies using this design generally require a follow-up period of at least 5 years, usually up to 10 years. In addition, they also need huge sample sizes to ensure statistically significant results. To obtain sufficient numbers of disease cases, a large number of healthy participants first have to be enrolled. Gene–environmental interaction analyses using genome-wide single nucleotide polymorphisms (SNPs) usually require 100,000–1,000,000 samples; to detect gene–environmental interactions in diabetes, stroke, or heart failure, which are associated with an annual morbidity rate of 0.2%, 200,000 people with 5-year follow-ups were needed which was calculated by the QUANTO program [1,7]. Currently, many large-scale prospective genomic cohort studies have been conducted or are being planned for worldwide, including the UK Biobank, Precision Medicine Initiative Cohort Program, and Tohoku Medical Mega Bank [8,9,10,11]. However, most of these studies have short follow-up times; thus, it can be considered that further studies with long-term follow-up data will allow us to detect novel gene–environmental interactions.

Another important approach for investigating gene–environmental interactions is Mendelian randomization, a method based on Mendel’s second law, the law of random assortment. Instead of directly using environmental factors, genetic markers, such as variants of SNPs, for which information can be obtained by GWASs, are used. Genetic information derived from germ cell lines is unchangeable during a person’s lifetime; thus, the possibility of reverse causation can be eliminated. In addition, due to the law of random assortment*,* this method can be a powerful tool when serious bias and/or confounding factors may affect the study results [12,13]. For example, in one previous meta-analysis investigating the association between alcohol intake and onset of cardiovascular disease [14], Mendelian randomization was conducted using variants of the alcohol dehydrogenase 1B gene (*ADH1B*). This study revealed that people with the enzyme variant related to low alcoholic decomposition activity tended to drink a lower amount of alcohol. In that study, the subjects could be divided into groups with high vs. low intake amounts based on Mendel’s second law. On the other hand, allocation to groups with low or high amounts of alcohol intake based on self-reported intake is extremely difficult, because it is impossible to limit the personal behavior for a long time, such as 10 years or more.

Another study investigating the association between consumption of isothiocyanate, abundantly present in vegetables, and the onset of lung cancer [15], used Mendelian randomization based on the genetic variants of the glutathione-*S*-transferase enzyme. This enzyme plays a role in the metabolism of isothiocyanate and the deactivation of polycyclic aromatic hydrocarbon, which is related to the risk of cancer onset. In this setting, adjustment for the affection of polycyclic aromatic hydrocarbon is needed; thus, exposure to polycyclic aromatic hydrocarbon, which is mainly found in cigarette smoke, was adjusted for by using the factor of smoking status as a co-variant in the multiple regression models or by conducting stratified analyses according to smoking status. This study suggested that Mendelian randomization can be a useful tool for investigating gene–environmental interactions. Nevertheless, this method has not been widely used. This is probably because the associations between genomic information and phenotypes, such as disease onset, will require future advances of genomic studies.

## 3. Significance of Gene–Environmental Interactions in Preventive Medicine

### 3.1. Applying Gene–Environmental Interaction Analyses to Preventive Medicine: Expectations and Limitations

Although prospective cohort studies are the most appropriate study design to clarify gene–environmental interactions with a high effect size, as mentioned in Section 2.2, long follow-up periods are required to obtain high-level evidence. Recently, the approach using GWASs, conducted using a case-control design, has been attempted in preventive medicine against NCDs. However, the adaptation of the findings to the development of preventive medicine has been limited. The main reason is that odds ratios of gene–environmental interactions usually range between 1–1.5, which is too small to use as benchmarks to conduct preventive interventions. For example, gene-environment interactions have been reported in breast cancer [16], with interactions between certain SNP variations and alcohol consumption reported to associate with increased breast cancer risk; however, the odds ratio was only 1.45 [17]. Currently, several research groups, including ours, are conducting studies that may lead to the development of effective preventive medicine in obesity.

### 3.2. Gene–Environmental Interactions in Obesity

The study by Speliotes et al. provided important information regarding gene–environmental interactions in obesity [18]. The authors reported 32 important SNPs associated with the risk of obesity in a European population and calculated the genetic risk score using information obtained from a GWAS by weighting each SNP according to the effect size. In brief, the genetic risk score was calculated using the β coefficients of the SNPs obtained from the previous GWAS [18,19]. Next, the genetic risk score was calculated by multiplying the number of effect alleles (0, 1, or 2) at each locus by the β coefficient of that SNP, dividing by the maximum allowable sum of the β coefficients, and then multiplying by twice the number of alleles. Higher scores indicate a greater genetic predisposition to obesity.

Using this score, handling genomic information of gene–environmental interactions may be facilitated. Accordingly, using this method, subsequent studies found an interaction between the genetic risk score and consumption of sugar-sweetened beverages in the development of obesity [20,21]. Individuals with a higher genetic risk score and consuming sugar-sweetened beverages showed significant increases in their body mass index (BMI) compared to those with lower genetic risk scores. Similar interactions have also been reported between BMI and fried foods [22] and between BMI and physical activities [23,24]. However, information on gene–environmental interactions in obesity is still limited, which is one of the main reasons for why an effective prevention method against obesity has not yet been established.

In our recent study, we elucidated the effects of gene-environment interactions on obesity, specifically between genetic factors and various obesity-related lifestyle factors, using data from a population-based prospective cohort study [25]. The genetic risk score from the β coefficients of 29 SNPs from East Asian subjects was used to conduct the analyses [18,19]. We reported several potentially useful gene–environmental interactions for the risk of high BMI, including associations between the genetic risk score and animal fat intake, vegetable fat intake, or animal protein intake. For example, in the group with a high genomic risk score, animal fat intake and fiber intake were significantly associated with BMI increases, while vegetable fat intake and animal protein intake were significantly associated with decreased BMI. On the other hand, in the group with the lowest genetic risk score, a high amount of carbohydrate intake and a sedentary lifestyle were associated with BMI increases. Thus, preventive measures in terms of lifestyle changes should be individualized according to the genomic risk, that is, personal preventive medicine. However, the sample size of our previous study was relatively small, with only approximately 1000 people included, and the statistical power was hence not sufficient to draw any definite conclusions. Validation studies using larger data sets are required in the future.

## 4. Challenges for Establishing Personalized Preventive Medicine

The use of large-scale prospective cohort studies would produce important findings in terms of gene–environmental interactions; however, some challenges remain to be discussed. First, “the missing heritability of complex disease” [26] has to be found. The genetic variants that have been found in GWASs can only explain a small proportion of the risk of disease onset. GWASs can effectively find common variants, with a frequency >5%, that are implicated in common diseases. However, low frequency variants, with a frequency of 0.01%–0.05% and with intermediate effects, cannot be found by GWASs [27,28], and this might account for a large part of “the missing heritability”. Thus, Manolio et al. [1,26] proposed several strategies to find this “missing heritability”. Their strategy focused on finding low-frequency variants with intermediate effects by various technical approaches, including thorough advances in sequencing. The latest technology in sequencing provides monumental increases in speed and volume, which could, in the future, allow us to find these variants by examining either the target region of interest or the whole genome [29].

These technologies will be of great benefit to gene–environmental interaction analyses using data from cohort studies. While the previous studies focused only on analysis using SNPs, which are common variants [18,25], gene–environmental analyses on the interactions of low-frequency variants and environmental factors will be able to be conducted in the future. Handling and analyzing the huge amounts of data obtained from the novel sequencing technologies will be another challenge in the near future. Although some methods have been proposed [30], future advances in data science will be required, and the progress made in artificial intelligence may be beneficial for this purpose.

Second, the strategies of applying the findings from gene–environmental interactions have to be considered. All information on gene–environmental interactions cannot be applied to the development of preventive medicine, even if statistically significant. Valid environmental factors to be intervened include personal lifestyle habits such as diet and exercise, which can be improved by personal efforts. Thus, developing preventive intervention programs aimed at improving such factors are possible, and primary prevention against developing a disease in the first place by improving personal lifestyle habits will continue to be the focus of preventive medicine. The next challenge will be to develop more effective, individualized programs. To investigate the effectiveness of various intervention programs, prospective studies comparing these programs are required. This phase will be essential for establishing effective personalized preventive medicine strategies.

Third, personalized preventive medicine is associated with higher costs than conventional preventive medicine. Gene typing costs much higher than conventional examinations, including blood tests, urine tests, radiography, cardiography, and physical examination. Measurement of genetic risk at the population level would be quite challenging in some countries. A person’s understanding and interpretation of genomic information is critical to conduct personalized preventive medicine using genomic information [31]. Therefore, education regarding “genetic literacy” is needed at least for patients who receive preventive interventions, which are associated with high additional costs. Thus, identification of the groups in which preventive medicine can be cost-effective, such as those who have a high risk for disease onset and in whom preventive intervention can effectively prevent this onset, is probably an appropriate approach to establish personalized preventive medicine.

## 5. Conclusions

Clarifying gene–environmental interactions in NCDs represents an attractive approach to establish personalized preventive medicine; however, there are currently limited data supporting interventions that can be easily adopted in clinical practice, while there are some promising preliminary studies. Longer follow-up studies of the present genomic prospective cohort studies will allow us to obtain practical evidence for the establishment of preventive medicine.

## Abbreviations

BMI	body mass index
GWAS	genome-wide association study
NCD	non-communicable disease
SNP	single nucleotide polymorphism
